# Genome-Wide Identification of QTL for Seed Yield and Yield-Related Traits and Construction of a High-Density Consensus Map for QTL Comparison in *Brassica napus*

**DOI:** 10.3389/fpls.2016.00017

**Published:** 2016-01-28

**Authors:** Weiguo Zhao, Xiaodong Wang, Hao Wang, Jianhua Tian, Baojun Li, Li Chen, Hongbo Chao, Yan Long, Jun Xiang, Jianping Gan, Wusheng Liang, Maoteng Li

**Affiliations:** ^1^Hybrid Rapeseed Research Center of Shaanxi Province, Shaanxi Rapeseed Branch of National Centre for Oil Crops Genetic ImprovementYangling, China; ^2^Department of Biotechnology, College of Life Science and Technology, Huazhong University of Science and TechnologyWuhan, China; ^3^Key Laboratory of Cotton and Rapeseed, Ministry of Agriculture, Institute of Industrial Crops, Jiangsu Academy of Agricultural SciencesNanjing, China; ^4^Institute of Biotechnology, Chinese Academy of Agricultural SciencesBeijing, China; ^5^Hubei Collaborative Innovation Center for the Characteristic Resources Exploitation of Dabie Mountains, Huanggang Normal UniversityHuanggang, China; ^6^Department of Applied Biological Science, College of Agriculture and Biotechnology, Zhejiang UniversityHangzhou, China

**Keywords:** *Brassica napus*, seed yield, seed yield-related traits, quantitative trait loci, map comparsion, candidate genes

## Abstract

Seed yield (SY) is the most important trait in rapeseed, is determined by multiple seed yield-related traits (SYRTs) and is also easily subject to environmental influence. Many quantitative trait loci (QTLs) for SY and SYRTs have been reported in *Brassica napus*; however, no studies have focused on seven agronomic traits simultaneously affecting SY. Genome-wide QTL analysis for SY and seven SYRTs in eight environments was conducted in a doubled haploid population containing 348 lines. Totally, 18 and 208 QTLs for SY and SYRTs were observed, respectively, and then these QTLs were integrated into 144 consensus QTLs using a meta-analysis. Three major QTLs for SY were observed, including *cqSY-C6-2* and *cqSY-C6-3* that were expressed stably in winter cultivation area for 3 years and *cqSY-A2-2* only expressed in spring rapeseed area. Trait-by-trait meta-analysis revealed that the 144 consensus QTLs were integrated into 72 pleiotropic unique QTLs. Among them, all the unique QTLs affected SY, except for *uq.A6-1*, including *uq.A2-3, uq.C1-2, uq.C1-3, uq.C6-1, uq.C6-5*, and *uq.C6-6* could also affect more than two SYRTs. According to the constructed high-density consensus map and QTL comparison from literatures, 36 QTLs from five populations were co-localized with QTLs identified in this study. In addition, 13 orthologous genes were observed, including five each gene for SY and thousand seed weight, and one gene each for biomass yield, branch height, and plant height. The genomic information of these QTLs will be valuable in hybrid cultivar breeding and in analyzing QTL expression in different environments.

## Introduction

*Brassica napus* (AACC, 2*n* = 38) originated from hybridization between *Brassica rapa* (AA, 2*n* = 20) and *Brassica oleracea* (CC, 2*n* = 18; UN, 1935), and is the second most important oilseed crop after soybean (Basunanda et al., 2010). As the global requirements for rapeseed oil and protein are growing rapidly, increasing seed yield (SY) is the main breeding aim at present. SY is directly determined by yield component traits, including thousand seed weight (SW), pod number per plant and seed number per pod (Qzer et al., 1999; Quarrie et al., 2006). In addition, SY is also indirectly influenced by other seed yield related traits (SYRTs), such as biomass yield (BY), plant height (PH), first effective branch height (BH), first effective branch number (FBN), length of main inflorescence (LMI), and pod number of main inflorescence (PMI) in *B. napus* (Qiu et al., 2006; Li et al., 2007; Shi et al., 2009). Interactions between SY, SW, PH, BH, FBN, LMI, and PMI were observed in previous studies (Yu, 1998; Zhang et al., 2006).

SY and SYRTs are all complex quantitative traits controlled by multiple genes (Kearsey and Pooni, 1998). QTL analysis has proved a powerful genetic approach to dissect complex traits (Paran and Zamir, 2003). Many QTLs for SY and SYRTs have been reported in *B. napus*, such as QTLs for SY being mainly located on A10, C3, and C6 (Quijada et al., 2006; Udall et al., 2006; Maccaferri et al., 2008). In addition, studies related to QTLs for SY and/or several SYRTs have also been performed (Chen et al., 2007, 2010; Li et al., 2007; Maccaferri et al., 2008; Shi et al., 2009; Basunanda et al., 2010; Ding et al., 2012; Cai et al., 2014). As the genetic backgrounds of different mapping populations for *B. napus* vary considerably, the number and location of QTLs detected in different populations also differ, thus is very necessary to contrast the QTLs for SY and SYRTs and select the common QTLs in different populations. Although many QTLs for SY and SYRTs have been reported, studies that simultaneously focused on the eight agronomic traits (SY, BY, SW, PH, BH, FBN, LMI, and PMI) are rare. Moreover, the candidate genes for these QTLs have rarely been mentioned. Comparative mapping among the model plant *Arabidopsis thaliana* with related species is a powerful tool to identify candidate genes. For example, Long et al. (2007) obtained the candidate gene *BnFLC10* underlying QTL *qFT10-4* and identified the key gene controlling differentiation of winter or spring type rapeseed based on comparative mapping analysis. Shi et al. (2009) and Ding et al. (2011) also obtained the candidate genes controlling flower time and seed phosphorus concentration, respectively, by comparative mapping with the *Arabidopsis* genome. Comparative mapping among *B. napus, Arabidopsis, B. rapa*, and *B. oleracea* genomes is necessary to obtain candidate genes in the confidence intervals (CIs) of QTLs for SY and SYRTs.

In order to increase statistical power and precision of obtaining QTLs, a high-density genetic linkage map is considered as a key factor (Jiang and Zeng, 1995). Several high-density genetic maps for *B. napus* have been constructed by integrating different linkage maps based on common molecular markers from different populations (Lombard and Delourme, 2001; Scoles et al., 2007; Raman et al., 2013). For example, Lombard and Delourme (2001) constructed a consensus map covering a total length of 2429.0 cM by integrating three individual linkage maps, and Wang et al. (2013) constructed a high-density consensus map with 1335 markers covering 2395.2 cM of the total genome length by merging eight individual linkage maps from different populations. Zhou et al. (2014) used 15 published articles concerning *B. napus* mapping experiments over the last decade and carried out *in silico* integration of 1960 QTLs with 13 SY and SYRTs, a total of 736 QTLs were mapped onto 283 loci in the A and C genomes of *B. napus*.

In the present study, a large doubled haploid (DH) population containing 348 lines was used to investigate the QTLs for SY and SYRTs in multiple environments, and then a consensus map was constructed for QTL comparison between the KN (the population used in this study) and five other published populations. These results provide abundant useful information to further understanding of the genetic mechanisms of SY and SYRTs, and could be used in marker assisted selection for improving SY in *B. napus*.

## Materials and methods

### Plant material and field experiments

A DH population, named KN and containing 348 lines derived from KenC-8 and N53-2, was used in this study (Wang et al., 2013). The KN genetic linkage map was constructed with 403 molecular markers, including 275 simple sequence repeats, 117 sequence-related amplified polymorphisms, 10 sequence tagged sites, and one intron fragment length polymorphism, which covered a total length of 1783.9 cM. The KN population and its parents were grown in eight environments, including a winter rapeseed area, Dali of Shannxi Province (coded DL), in northwest China for five successive years (September–May of 2008–2009, 2009–2010, 2010–2011, 2011–2012, and 2012–2013); a spring rapeseed area, Sunan of Gansu Province (coded GS), in northwest China for three successive years (April–September of 2010, 2011, and 2012). Year-location combinations were treated as micro-environments, for example, 09DL means that the experiment was carried out in September–May of 2009–2010 at DL. Meanwhile, each year-location combination was treated as a trial.

The field experiments followed a randomized complete block design. The KN population, together with the two parents, was planted in DL and GS with three and two replications, respectively. Each field trial consisted of 348 lines. Each line was grown in a two-row plot with 40 cm between rows and 20 cm between individuals, and row length of 250 cm in all environments.

### Measurement of phenotypic data of SY and SYRTs

Phenotypic data for SY (g/plant) were recorded with five representative plants in the middle of each plot. These five plants were also used for measurement of other SYRTs: BY (g/plant), SW (g), PH (cm), BH (cm), FBN, LMI (cm), and PMI. Because of a strong requirement for vernalization, the N53-2 and some DH lines did not flower or fully mature in the spring area (10GS, 11GS, and 12GS), and so SY of these DH lines were treated as missing data. SY was the average dry weight of seeds of the five representative individuals. BY was measured as the average total above-ground dry weight of the five plants (excluding the seeds). SW was the average dry weight of 1000 well-filled seeds from the five samples. PH was the average height of the five individuals, measured from the base of the stem to the tip of the main inflorescence. FBN was the number of branches arising from the main stem of each harvested individual. LMI was measured from the bottom to the top of the main inflorescence. PMI was effective pod number from the bottom to the top of the main inflorescence.

### Statistical analysis, QTL mapping and meta-analysis

Estimates of means and variances for the SY and SYRTs were implemented using SPSS 18.0 software (SPSS Inc., Chicago, IL, USA). The QTL information for PH was according to Wang et al. (2015) in 10DL, 11DL, 12DL, 11GS, 12GS, and 12WH. QTL detection for other traits was conducted by composite interval mapping with Windows QTL Cartographer 2.5 software (Wang et al., 2007). The estimated additive effect and phenotypic variation explained (PVE) by each putative QTL were obtained using composite interval mapping model. Significance levels for the log odds score methods (LOD) were determined by 1000-permutation test corresponding to *P* = 0.05, and LOD of 2.8–3.1 was used to, respectively, identify significant QTLs in each environment, and these QTLs were termed “identified QTL.” QTLs that mapped to the same region with overlapping CIs were assumed to be the same, and BioMercator 2.1 software was used to integrate these QTLs into consensus QTLs using the meta-analysis method (Arcade et al., 2004). If a consensus QTL had at least one environment with PVE ≥ 20% or at least two environments with PVE ≥ 10%, the QTL was defined as a major QTL; the remaining QTLs were defined as minor QTLs (Maccaferri et al., 2008).

The identified QTLs for SY and SYRTs were named according to Wang et al. (2013); for example, the QTL abbreviation “*qSY*” (*q*, QTL; *SY*, seed yield) suffixed with the linkage group (A1–A10, C1–C9), a hyphen (-), and finally the serial number of QTLs in the linkage group (e.g., *qSY-A2-1*). The QTL integrations were adopted by meta-analysis; for example, the identified QTLs were integrated into consensus QTLs trait-by-trait, and the consensus QTLs for SY and SYRTs with overlapping CIs were integrated into pleiotropic unique QTLs using BioMercator 2.1 (Arcade et al., 2004). The name of consensus QTLs and unique QTLs referred to the name of identified QTLs. For each unique QTL, one or more consensus QTLs for SYRTs were chosen as indicator QTLs, which were defined as potential genetic determinants of the co-localized QTL for SY.

### QTL projection from other populations onto the KN map, and QTL comparison among the different populations

The map projection package of BioMercator 2.1 software was used for QTL projection of SY and SYRTs in five previously reported populations onto the KN genetic map (Table 1), including the QN (Quantum × No. 2127-17; Chen et al., 2007), SE (SI-1300 × Eagle; Li et al., 2007), ER (Express617 × R53; Radoev et al., 2008), TN (Tapidor × Ningyou7; Shi et al., 2009), and BE (B104-2 × Eyou Changjia; Ding et al., 2012) populations. The method for projection of QTLs from different linkage groups was according to Arcade et al. (2004). The method for QTL comparison from different linkage groups was a “two-round” strategy (Shi et al., 2013), and the detailed methods for QTL comparison from different populations were found in Ding et al. (2012) and Jiang et al. (2014). The consensus QTLs from different populations were named with the population abbreviation followed by the consensus QTL names for QTL comparison (e.g., *KNcqSY-A2-1*).

**Table 1:** **Five reported populations were used for QTL projection of SY and SYRTs in ***B. napus*****.

**Population parental lines**	**Abbreviation**	**Size**	**Type**	**Traits**	**References**
Tapidor × Ningyou7	TN	202	DH	BN BY PH SW SY	Shi et al., 2009
SI-1300 × Eagle	SE	184	F_2_	PH HPB ELMI SMI	Li et al., 2007
B104-2 × Eyou Changjia	BE	124	F_2_	SY SW BN PH	Ding et al., 2012
Express617 × R53	ER	250	DH	SY SW	Radoev et al., 2008
Quantum × No. 2127-17	QN	258	DH	PH HPB LMI FB	Chen et al., 2007

### Candidate gene observations by comparative mapping among *Arabidopsis, B. rapa, B. oleracea, and B. napus*

Among the 403 markers mapped in the KN genetic map (Table S1), 141 markers with known sequence information were used for the sequence comparisons of the *Arabidopsis* genome database with other *Brassica species* (http://www.arabidopsis.org/). The databases of *B. oleracea* (Liu et al., 2014), *B. rapa* (http://brassicadb.org/brad/), and *B. napus* (http://www.genoscope.fr/brassicanapus/) were used for confirmation of homologous genes on the genomes of *Arabidopsis, B. rapa, B. oleracea*, and *B. napus*. Firstly, the 141 markers with known sequence information were used as anchored markers to carry out map alignment between *B. napus* and *Arabidopsis* according to the method of Long et al. (2007). If three or more sequence informative markers in the KN population were closely linked within one conserved block of *Arabidopsis* (Schranz et al., 2006), a synteny block was considered to exist. If there were only one or two sequence informative marker(s), this was recognized as an insertion segment. Secondly, if a synteny block or insertion segment were co-localized with the CI of a QTL, the genes underlying the synteny block or insertion segment were considered as candidate genes for the QTL. Thirdly, the genes of *Arabidopsis* were used to identify homologous genes in *B. rapa, B. oleracea*, and *B. napus*. The detailed methods are found in Long et al. (2007) and Shi et al. (2009).

### Ethical standards

The authors declare that the experiments comply with the current laws of the country in which they were performed.

## Results

### Phenotypic analysis and genetic correlation between SY and SYRTs

**Figure 1:**
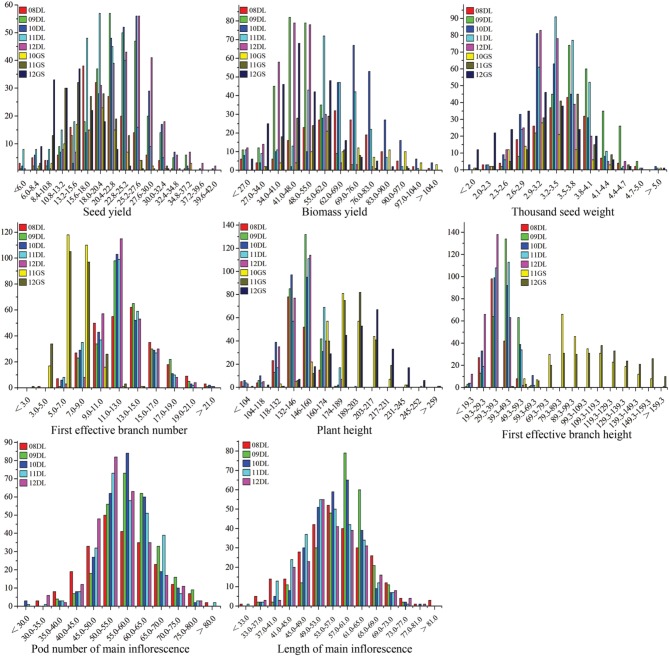
**The frequency distribution of SY and SYRTs in multiple environments**. The units of the x-axis are the phenotypic values, and the units of the y-axis are the number of lines. SY and SYRTs in different experiments was discriminated using different colored boxes. A unit of measurement: seed yield (g), biomass yield (g), thousand seed weight (g), plant height (cm), first effective branch height (cm), first effective branch number, length of main inflorescence (cm), pod number of main inflorescence.

The SY and SYRTs of the two parents and the KN population showed differences in most micro-environments (Table 2). There was a wide segregation range of SY, with a continuous normal distribution and transgressive segregation in all trials (Figure 1), suggesting that SY was a quantitative trait with polygenic control. Seven other SYRTs (BY, SW, PH, BH, FBN, LMI, and PMI) also showed a wide segregation range in all trials with normal or near-normal distributions.

**Table 2:** **Mean values and phenotypic variation for SY and SYRTs of KN population evaluated in different micro-environments**.

**SY(g)^a^**		**08DL^c^**	**09DL**	**10DL**	**10GS**	**11DL**	**11GS**	**12DL**	**12GS**
KenC-8	Mean^b^	20.08 ± 1.82	24.65 ± 4.81	24.25 ± 6.80	24.68 ± 1.22	16.22 ± 0.91	22.37 ± 1.73	18.77 ± 2.03	23.23 ± 3.63
N53-2	Mean	27.00 ± 4.59	24.23 ± 4.91	25.09 ± 4.41		25.29 ± 2.86		29.18 ± 3.50	
DH	Mean	19.48 ± 5.38	22.29 ± 5.13	22.87 ± 5.74	18.47 ± 5.59	18.76 ± 5.46	16.91 ± 5.15	23.83 ± 5.45	14.36 ± 4.25
Range	Min-Max	2.24–34.91	5.96–35.07	4.3–38.82	8.1–35.1	2.45–34.81	6.43–33.74	6.04–40.83	4.12–26.37
**BY(g)**		**08DL**	**09DL**	**10DL**	**10GS**	**11DL**	**11GS**	**12DL**	**12GS**
KenC-8	Mean	58.27 ± 4.49	74.49 ± 20.09	70.69 ± 18.56	106.63 ± 14.95	59.99 ± 5.31	91.66 ± 15.33	39.49 ± 5.11	57.09 ± 1.60
N53-2	Mean	86.74 ± 13.92	71.04 ± 14.84	76.92 ± 12.52		71.37 ± 6.75		45.81 ± 8.43	
DH	Mean	62.68 ± 17.14	46.44 ± 9.83	71.08 ± 16.04	84.49 ± 19.28	59.52 ± 14.78	53.03 ± 11.56	45.61 ± 9.62	48.08 ± 13.54
Range	Min–Max	9.7–107.1	17.1–74.0	16.4–108.2	41.8–135.7	12.9–97.9	27.9–95.0	14.0–116.6	14.0–116.6
**SW(g)**		**08DL**	**09DL**	**10DL**	**10GS**	**11DL**	**11GS**	**12DL**	**12GS**
KenC-8	Mean	3.53 ± 0.31	3.72 ± 0.31	3.34 ± 0.43	3.29 ± 0.01	2.70 ± 0.50	3.15 ± 0.54	3.00 ± 0.49	3.15 ± 0.54
N53-2	Mean	3.93 ± 0.10	4.02 ± 0.29	3.47 ± 0.47		2.88 ± 0.23		3.33 ± 0.12	
DH	Mean	3.43 ± 0.55	3.76 ± 0.47	3.31 ± 0.51	3.15 ± 0.52	2.81 ± 0.52	3.45 ± 0.56	3.3 ± 0.46	2.92 ± 0.72
Range	Min–Max	1.48–4.83	2.47–4.92	1.91–5.71	1.73–5.20	1.57–5.78	2.00–5.93	2.24–5.64	1.11–5.24
**PH(cm)**		**08DL**	**09DL**	**10DL**	**10GS**	**11DL**	**11GS**	**12DL**	**12GS**
KenC-8	Mean	120.00 ± 10.00	144.00 ± 6.56	145.67 ± 6.03	172.45 ± 7.84	151.67 ± 11.59	191.00 ± 1.41	135.67 ± 9.07	208.50 ± 6.36
N53-2	Mean	145.00 ± 13.23	156.33 ± 13.43	166.00 ± 5.57	176.87 ± 10.78	165.33 ± 11.93	181.50 ± 27.58	156.67 ± 11.06	228.00 ± 7.07
DH	Mean	128.29 ± 16.64	148.66 ± 16.97	143.28 ± 18.49	185.18 ± 24.86	152.52 ± 19.36	189.31 ± 30.44	132.87 ± 17.64	196.95 ± 25.03
Range	Min–Max	83.3–163.3	90.0–175.7	94.3–182.0	108.0–249.1	94.7–193.0	132.0–296.5	87.3–170.3	103.0–261.2
**BH(cm)**		**08DL**	**09DL**	**10DL**	**11DL**	**11GS**	**12DL**	**12GS**	
KenC-8	Mean	27.04 ± 2.25	47.47 ± 5.01	36.20 ± 10.11	53.60 ± 4.13	95.50 ± 10.89	33.87 ± 6.62	101.45 ± 3.54	
N53-2	Mean	39.00 ± 7.21	58.13 ± 5.67	54.87 ± 7.51	54.20 ± 5.64	122.60 ± 3.96	43.27 ± 6.20	184.74 ± 9.85	
DH	Mean	26.82 ± 6.98	43.02 ± 8.74	39.65 ± 10.85	54.57 ± 8.36	101.31 ± 24.09	33.07 ± 7.67	113.43 ± 27.64	
Range	Min–Max	4.3–52.1	3.6–66.3	3.5–65.1	14.3–74.9	47.7–180.8	2.9–51.4	49.0–187.8	
**FBN**		**08DL**	**09DL**	**10DL**	**11DL**	**11GS**	**12DL**	**12GS**	
KenC-8	Mean	13.4 ± 1.6	17.9 ± 1.7	14.7 ± 1.7	16.9 ± 0.6	8.3 ± 1.5	13.6 ± 1.4	10.5 ± 1.2	
N53-2	Mean	11.9 ± 0.9	10.4 ± 1.6	9.5 ± 0.8	8.7 ± 0.8	6.3 ± 0.7	9.4 ± 2.3	6.4 ± 1.1	
DH	Mean	14.3 ± 4.42	11.1 ± 1.73	11.3 ± 2.30	115.0 ± 2.23	7.8 ± 1.57	10.1 ± 1.58	7.2 ± 1.72	
Range	Min–Max	6.3–25.5	5.4–19.2	5.5–22.9	5.2–20.6	3.4–13.4	4.5–15.3	2.8–14.5	
**LMI(cm)**		**09DL**	**10DL**	**11DL**	**12DL**				
KenC-8	Mean	46.13 ± 2.62	51.94 ± 2.32	42.62 ± 5.63	50.92 ± 3.32				
N53-2	Mean	60.92 ± 5.38	65.97 ± 1.72	67.94 ± 3.45	69.54 ± 3.78				
DH	Mean	58.09 ± 6.99	55.75 ± 6.87	53.64 ± 8.02	55.27 ± 9.69				
Range	Min-Max	35.0–79.5	34.1–78.1	32.1–74.7	33.8–79.7				
**PMI**		**09DL**	**10DL**	**11DL**	**12DL**				
KenC-8	Mean	55.2 ± 1.8	57.2 ± 2.9	59.0 ± 3.7	49.7 ± 7.2				
N53-2	Mean	62.5 ± 4.8	66.6 ± 1.2	73.7 ± 3.0	61.7 ± 5.4				
DH	Mean	59.7 ± 8.1	57.4 ± 7.7	63.2 ± 9.1	55.4 ± 8.5				
Range	Min–Max	36.5–88.3	23.7–80.5	23.9–93.2	30.4–77.6				

a *Seed yield and related traits in different microenvironments*.

b *Mean value ± SD*.

c *Micro-environments*.

“*KenC-8” represents male parent, “N53-2” represents female parent*.

*SY, seed yield; BY, biomass yield; SW, thousand seed weight; PH, plant height; BH, first effective branch height; FBN, first effective branch number; LMI, length of main inflorescence; PMI, pod number of main inflorescence*.

The correlations between SY and SYRTs showed large differences (Table 3). The results indicated that SY was highly and positively correlated with SYRTs except for FBN, and especially for BY with a correlation coefficient of 0.83. LMI was significantly positively correlated with SY and SYRTs except for FBN. SW was significantly positively correlated with SY (0.31), BY (0.53), PH (0.42), and LMI (0.37). The high correlations among SY and SYRTs indicated that these traits might be controlled by the same kinds of genes in some cases.

**Table 3:** **Pearson correlation coefficients for trait pairs affecting SY and SYRTs in KN population**.

**Trait**	**SY**	**BY**	**SW**	**PH**	**BH**	**FBN**	**LMI**	**PMI**
SY	1	0.83^**^	0.31^**^	0.60^**^	0.37^**^	0.07	0.41^**^	0.49^**^
BY	0.83^**^	1	0.53^**^	0.74^**^	0.39^**^	0.22^**^	0.53^**^	0.37^**^
SW	0.31^**^	0.53^**^	1	0.42^**^	0.11	0.06	0.37^**^	0.11
PH	0.60^**^	0.74^**^	0.42^**^	1	0.69^**^	0.12^*^	0.74^**^	0.39^**^
BH	0.37^**^	0.39^**^	0.11	0.69^**^	1	0.03	0.32^**^	0.33^**^
FBN	0.07	0.22^**^	0.06	0.12^*^	0.03	1	−0.22^**^	−0.02
LMI	0.41^**^	0.53^**^	0.37^**^	0.74^**^	0.32^**^	−0.22^**^	1	0.35^**^
PMI	0.49^**^	0.37^**^	0.11	0.39^**^	0.33^**^	−0.02	0.35^**^	1

* *p < 0.05*,

** *p < 0.01, respectively*.

### Genome-wide QTL detection for SY and SYRTs

**Figure 2:**
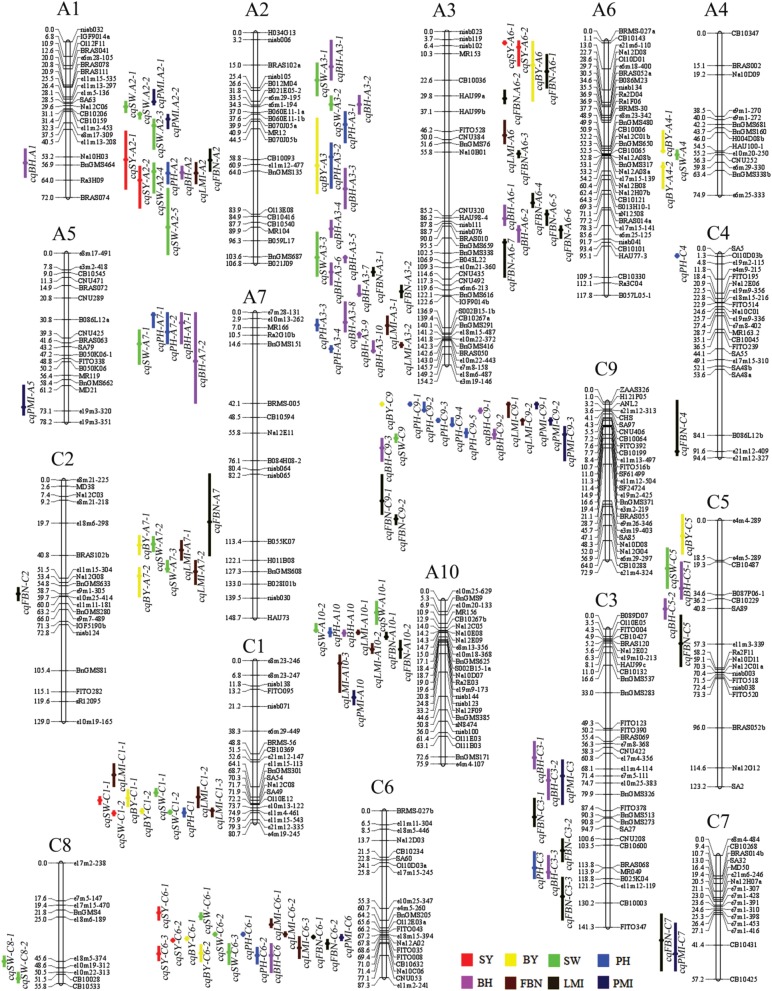
**Genetic linkage map and the location of QTLs for SY and SYRTs in the KN linkage map**. The 144 consensus QTLs for SY and SYRTs were distributed on 18 linkage groups with the exception of A8, A1–A10 were represented by the A genome and C1–C9 were represented by the C genome in *B. napus*. The loci names were listed on the right of the linkage groups, while position of loci were showed on the left side of linkage groups. The consensus QTLs associated with SY and SYRTs were indicated by bars with various backgrounds on the left of each linkage group (Red bar, seed yield; Yellow bar, biomass yield; Green bar, thousand seed weight; Blue bar, plant height; Purple bar, first effective branch height; Brown bar, first effective branch number; Black bar, length of main inflorescence; Dark blue bar, pod number of main inflorescence).

In total, 226 identified QTLs were observed for SY and SYRTs: 18 for SY and 208 for SYRTs (Table S2). These 226 QTLs were integrated into 144 consensus QTLs, which were located on 18 linkage groups with the exception of A8 (Figure 2, Figure S1).

For SY, there were nine consensus QTLs obtained, mainly located on A2, A6, C1, and C6 (Figure 2, Table S2). Four of these QTLs were repeatedly detected in different experiments, including *cqSY-C1-2* and *cqSY-C6-2*, detected in four successive years in DL (09DL, 10DL, 11DL, and 12DL), and *cqSY-C6-3* detected in three experiments (09DL, 10DL, and 11DL). In addition, both *cqSY-C6-2* and *cqSY-C6-3* were assumed to be major QTLs with PVE > 10% in two environments. Meanwhile, *cqSY-A2-2*, which was only observed in 10GS, was also a major QTL with PVE = 20.91% (Table 4).

**Table 4:** **Detailed information of seven major QTLs for SY and SYRTs in the KN population**.

**Consensus QTLs**	**Peak (cM)**	**CI^a^(cM)**	**Identified QTLs**	**Chr.^b^**	**LOD**	**A^c^**	**PVE^d^**	**E^e^**
*cqSY-A2-2*	68.11	61.0–81.2	*qSY-10GS2*	A2	5.55	−17.04	20.91	10GS
			*qSY-09DL16-1*	C6			5.63	09DL
*cqSY-C6-2*	70.06	69.33–70.79	*qSY-10DL16-1*	C6	7.40	9.53	11.69	10DL
			*qSY-12DL16-2*	C6			9.04	12DL
			*qSY-11DL16-1*	C6			13.13	11DL
*cqSY-C6-3*	76.14	72.85–79.42	*qSY-09DL16-2*	C6	8.78	10.06	6.76	09DL
			*qSY-10DL16-2*	C6			14	10DL
			*qSY-11DL16-2*	C6			15.92	11DL
*cqSW-A7-2*	114.65	111.12–118.18	*qSW-11DL7*	A7	6.9	0.16	9.99	11DL
			*qSW-09DL7-2*	A7			6.16	09DL
			*qSW-10DL7*	A7			11.92	10DL
			*qSW-12DL7-1*	A7			13.95	12DL
*cqSW-C1-1*	66.68	64.83–68.54	*qSW-08DL11-1*	C1	6.6	−0.17	19.62	08DL
			*qSW-11DL11-1*	C1			4.58	11DL
			*qSW-08DL11-2*	C1			17.83	08DL
			*qSW-09DL11-1*	C1			5.52	09DL
			*qSW-10DL11-1*	C1			5.73	10DL
*cqBH-A2*	64.69	61.71–67.68	*qBH-11GS2*	A2	12.7	10.45	14.58	11GS
			*qBH-12GS2*	A2			17.17	12GS
*cqFBN-C6-1*	68.6	67.86–69.35	*qFBN-09DL16*	C6	13.04	−0.91	19.14	09DL
			*qFBN-10DL16*	C6			13.47	10DL

a *Confidence interval*.

b *Chromosome*.

c *Additive*.

d *Phenotypic variation explained by each identified QTL*.

e *The environment in which QTL were detected*.

*DL, Dali; GS, Sunan; 08, 09, 10, 11, and 12 indicated the years of 2008, 2009, 2010, 2011, and 2012, respectively. The QTL abbreviation “q” represents identified QTL, the QTL abbreviation “cq” represents consensus QTL*.

For SYRTs, the number of QTLs for different traits clearly differed. For BY, 13 consensus QTLs were obtained, mainly located on A7, C1, and C6 with 4.34–19.96% of PVE (Figure 2, Table S2). Five of these QTLs were repeatedly detected in different experiments, for example, *cqBY-C1-2, cqBY-C6-1*, and *cqBY-C6* were detected in three experiments.

For SW, 25 consensus QTLs were detected, distributed on 11 linkage groups (Figure 2, Table S2). Among them, nine consensus QTLs were repeatedly detected in different environments (Table S3). QTLs *cqSW-A7-2, cqSW-C1-1*, and *cqSW-C1-2* were repeatedly detected in four experiments in winter area—with *cqSW-A7-2* and *cqSW-C1-1* regarded as two major QTLs with PVE > 10% in two environments (Table 4). The QTL *cqSW-C9-1* that appeared in both winter and spring areas was an insensitive QTL in terms of response to environments.

For PH, 18 consensus QTLs were obtained at the mature stage in eight environments (Wang et al., 2015), and mainly located on A3, C6, and C9 (Figure 2, Table S2). Seven QTLs were repeatedly detected in different environments (Table S3), including *cqPH-A3-3* detected in six environments, and *cqPH-C6-2* and *cqPH-C9-5* detected in three environments (Wang et al., 2015).

For BH, 27 consensus QTLs were integrated from 41 identified QTLs, and mainly located on A2, A3, A10, and C9 (Table S2). Ten QTLs were repeatedly detected in different environments (Table S3), for example, *cqBH-A3-2* and *cqBH-A10* were repeatedly observed both in winter and spring areas. Additionally, QTL *cqBH-A2* was regarded as a major QTL with PVE > 10% in 11GS and 12GS. Because BH was highly positively correlated with PH, the major QTL *cqBH-A2* for BH might also regulate PH.

For FBN, 25 consensus QTLs were obtained and were located on 12 chromosomes (Table S2). Three QTLs were repeatedly detected in both winter and spring areas: *cqFBN-A3-1, cqFBN-A3-2*, and *cqFBN-C3-2*. In addition, *cqFBN-A2* and *cqFBN-C2* were repeatedly detected in two and three experiments, respectively. One important QTL, *cqFBN-C6-1* with PVE > 10% in two environments, was considered as a major QTL (Table 4).

For LMI, 24 identified QTLs were detected and integrated into 17 consensus QTLs, of which four consensus QTLs were repeatedly detected in different experiments (Table S2). For example, *cqLMI-A3-1* and *cqLMI-A3-2* were repeatedly detected in three and four experiments, respectively. However, no major QTL was observed for LMI. For PMI, 10 consensus QTLs were obtained and only two were repeatedly detected in different environments: *cqPMI-A10* and *cqPMI-C6* in spring (11GS and 12GS) and winter areas (09DL and 11DL), respectively. No QTLs for PMI reached the standard of a major QTL.

**Figure 3:**
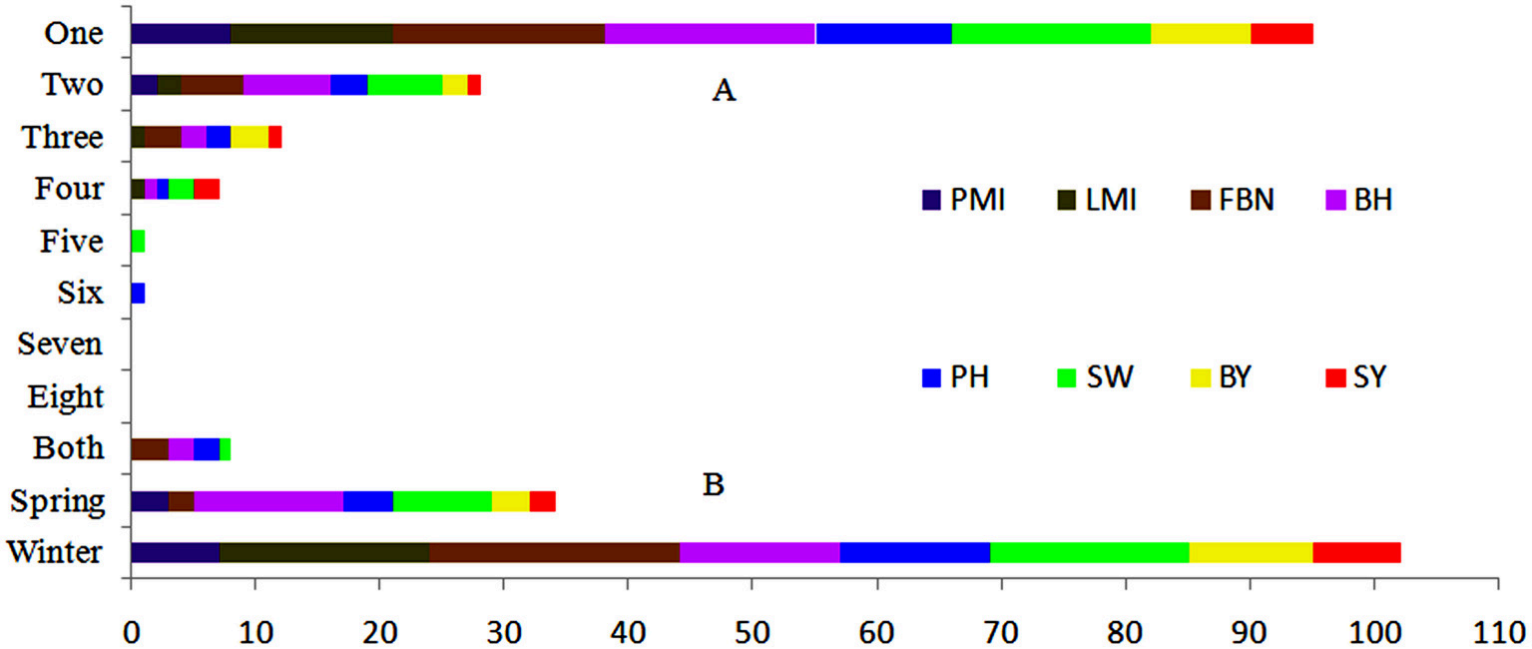
**Expression response of 144 consensus QTLs in nature environments. (A)** Number of consensus QTLs appeared in one to eight micro-environments. **(B)** Number of consensus QTLs appeared in winter, spring or both macro-environments.

In conclusion, with 22, linkage group A3 had the largest number of consensus QTLs, followed by C6 and C9, both with 17. More than half of the consensus QTLs (95 of 144) for SY and SYRTs were detected in one micro-environment (Table S3). Otherwise, 28, 13, and 7 consensus QTLs were identified in two, three and four micro-environments, respectively (Figure 3). In total, 102 and 34 consensus QTLs were detected in winter (DL) and spring areas (GS), respectively, and only eight appeared in both areas (Figure 3, Table S3). These results indicated that the majority of consensus QTLs were expressed principally in response to a specific environment.

### The unique QTL analysis for SY and SYRTs

Of the 144 consensus QTLs for SY and SYRTs, 112 QTLs with overlapping CIs were integrated into 40 unique QTLs, and the remaining 32 consensus QTLs were only detected for one trait (Table S4). Altogether, 72 unique QTLs were obtained, in which 39 unique QTLs, respectively, affected 2–6 different traits. These unique QTLs were considered as pleiotropic (Table S4), such as *uq.A2-3* which was integrated from two QTLs for SY and five for SYRTs. Notably, *uq.A2-3, uq.C6-5*, and *uq.C6-6* were integrated from 3 to 6 QTLs of different traits and contained three major QTLs for SY (Table 5).

**Table 5:** **Detailed information of unique QTLs for SY in KN population**.

**Unique QTLs**	**Position (cM)**	**CI (cM)**	**Consensus QTLs**	**Chr**.	**Traits**	**A**
*uq.A2-3*	63.95	62.0–65.9	*cqSY-A2-1*	A2	SY	−8.987
			*cqSW-A2-4*	A2	SW	−0.262
			*cqFBN-A2*	A2	FBN	−0.416
			*cqLMI-A2*	A2	LMI	1.975
			*cqPH-A2*	A2	PH	7.587
			*cqBH-A2*	A2	BH	10.449
			*cqSY-A2-2*	A2	SY	−17.037
*uq.A6-1*	0.03	0.0–0.6	*cqSY-A6-1*	A6	SY	8.928
			*cqSY-A6-2*	A6	SY	6.441
*uq.C1-2*	68.58	67.4–69.8	*cqLMI-C1-2*	C1	LMI	−1.742
			*cqSW-C1-1*	C1	SW	−0.173
			*cqBY-C1-1*	C1	BY	−25.009
			*cqSY-C1-1*	C1	SY	−7.728
*uq.C1-3*	75.67	75.1–76.3	*cqPH-C1*	C1	PH	−2.997
			*cqLMI-C1-3*	C1	LMI	−1.561
			*cqBY-C1-2*	C1	BY	−17.639
			*cqSW-C1-2*	C1	SW	−0.152
			*cqSY-C1-2*	C1	SY	−7.242
*uq.C6-1*	58.5	57.0–60.0	*cqSY-C6-1*	C6	SY	7.459
			*cqSW-C6-1*	C6	SW	0.150
*uq.C6-5*	70.23	69.5–70.9	*cqLMI-C6-3*	C6	LMI	1.899
			*cqFBN-C6-2*	C6	FBN	−0.637
			*cqSY-C6-2*	C6	SY	9.530
*uq.C6-6*	75.93	73.7–78.2	*cqSW-C6-3*	C6	SW	0.119
			*cqBY-C6-2*	C6	BY	21.017
			*cqSY-C6-3*	C6	SY	10.062

*The QTL abbreviation “cq” represents consensus QTL, the QTL abbreviation “uq” represents unique QTL*.

There were 21 unique QTLs observed, which were integrated from two consensus QTLs that controlled different traits (Table S4). For example, *uq.A3-1, uq.A3-2, uq.A3-6*, and *uq.C5-2* all included QTLs for SW and BH. Likewise, *uq.A2-1* and *uq.A2-2* were composed of QTLs for SW and PMI. Although *uq.A10-3* was a pleiotropic QTL and was integrated from three consensus QTLs, it was only closely linked to FBN and LMI. In addition, *uq.A4-1* controlled BY and *uq.C6-1* controlled SY, which were both closely linked to the QTL for SW. These results also explained why the QTL for SW was closely linked to the QTL for SY or BY.

Some unique QTLs were integrated from QTLs for more than two SYRTs (Table S5). QTL *uq.A3-4* controlled BY, *uq.A3-9*, and *uq.A3-11* controlled LMI, and *uq.A7-2* controlled SW, which were all, respectively, tightly linked to the BH and PH. *Uq.A7-3* controlled FBN and *uq.C1-2* controlled SY, which were both respectively closely linked to BY, SW, and LMI. It is noteworthy that *uq.A10-2* and *uq.C1-3* were closely related to five SYRTs; *uq.A10-2* controlled BH and FBN, and *uq.C1-3* controlled SY and BY, which were both tightly linked to SW, PH, and LMI.

### Consensus map construction and QTL comparison for SY and SYRTs among different mapping populations

**Figure 4:**
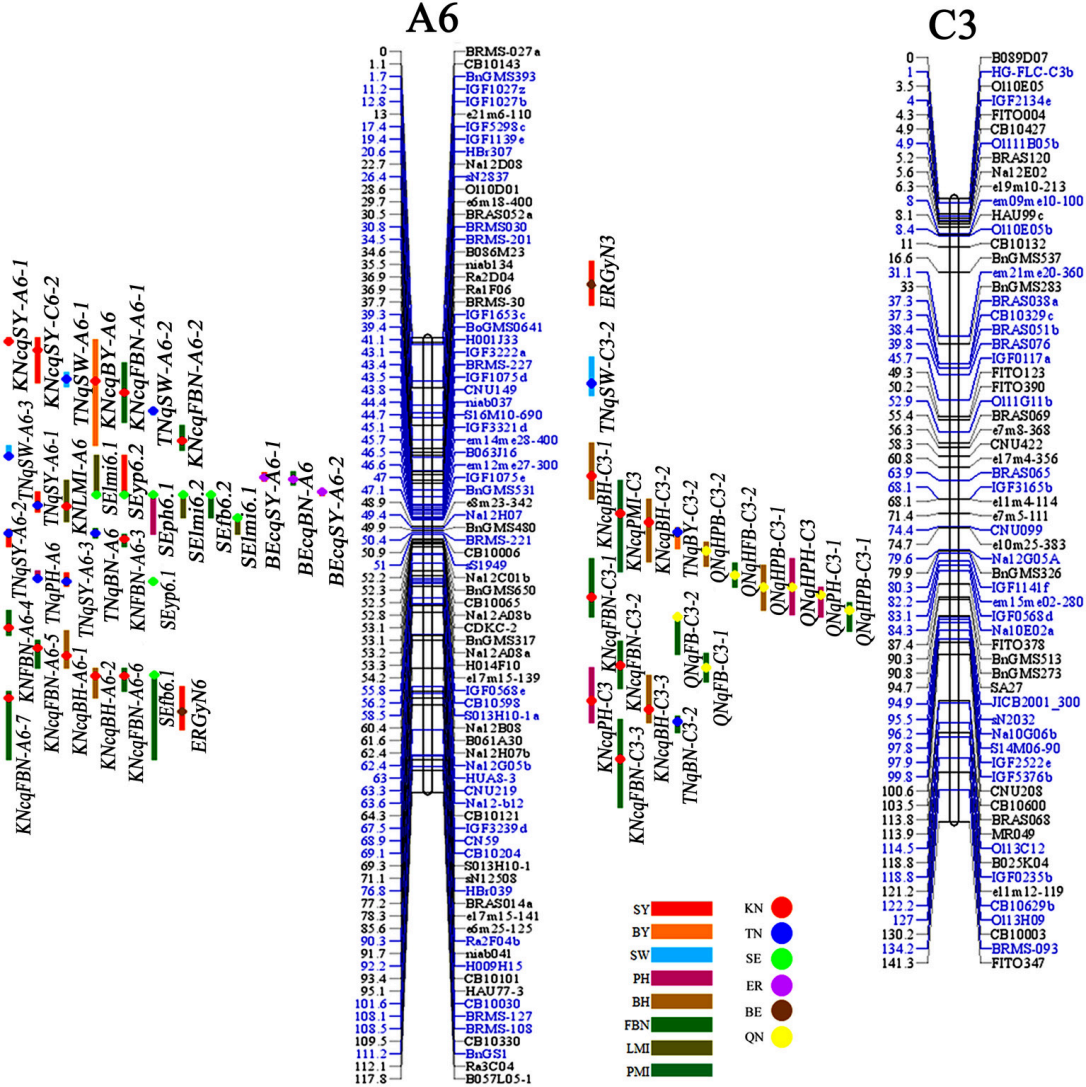
**The consensus map and QTLs for SY and SYRTs in different populations (A6 and C3)**. Markers with blue color indicated these makers were projected from other maps on the KN map based on common markers by BioMercator 2.1 software. QTLs for SY and SYRTs detected in different populations were discriminated with different color bars on the left of each linkage group. Red bar, SY (seed yield); Orange bar, BY (biomass yield); Cambridge blue bar, SW (thousand seed weight); Purple bar, PH (plant height); Claybank bar, BH (first effective branch height); Green bar, FBN (first effective branch number); Breen bar, LMI (length of main inflorescence); Blue-green bar, PMI (pod number of main inflorescence). The KN population and five populations were indicated by disks with various backgrounds on the bars of each QTL. Red disk, KN population; Blue disk, TN population; Light green disk, SE population; Light purple disk, ER population; Brown disk, BE population; Yellow disk, QN population.

In the present study, five published populations (QN, SE, ER, TN, and BE) for QTL analysis of SY and SYRTs were used for consensus map construction and QTL comparison (Table 1, Table S6). QTLs collected in each population were first integrated into consensus QTLs using BioMercator 2.1 software. A high-density consensus map with 907 molecular markers was constructed (Figure S3). A total of 480 consensus QTLs for SY and SYRTs from five populations were obtained and 166 consensus QTLs were successfully projected onto the consensus map (Figure 4, Figures S2, S3).

**Figure 5:**
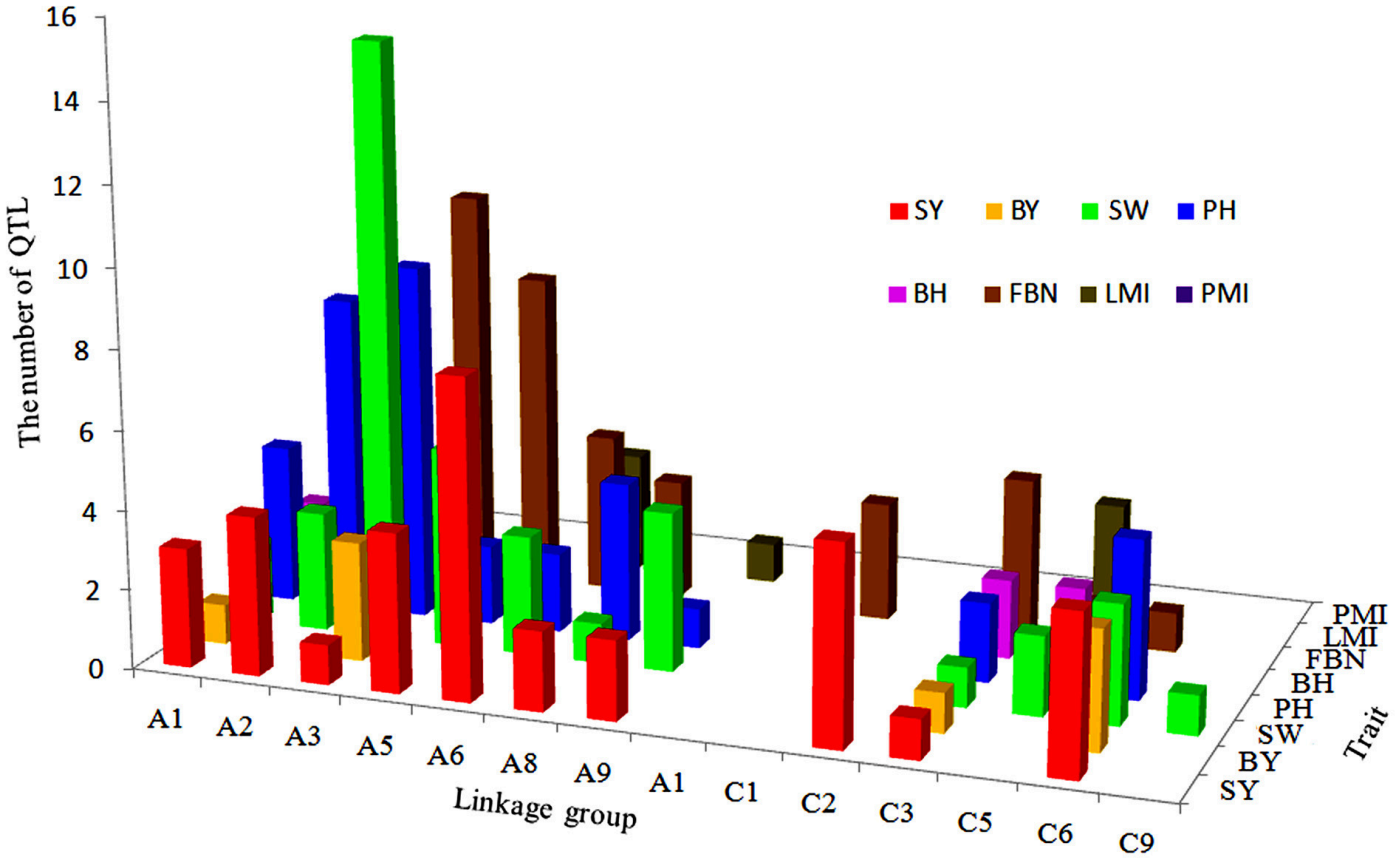
**The number and distribution of projection QTLs for SY and SYRTs from five population in ***B. napus*****. The x-axis of abscissas indicated 19 linkage groups, y-axis indicated the number of projection QTLs and z-axis indicated SY and SYRTs.

A total of 34 QTLs for SY were projected onto the KN consensus map, half of which were located on A2, A5, A6, C2, and C6 (Figure 5, Figure S3, Table S7). This revealed that *KNcqSY-A2-2, KNcqSY-C6-1*, and five QTLs of the TN population (*TNqSY-A2-2, TNqSY-A2-3, TNqSY-A2-4, TNqSY-C6-1*, and *TNqSY-C6-3*) were co-localized on A2 and C6, respectively. The results indicated that these QTLs might have some important genes for SY and could be expressed stably in different genetic backgrounds. In addition, five ortholougs genes (*GASA4, ATCLH1, RBCS1A, LQY1*, and *ATGGH1*) for SY were aligned in the CIs of these QTLs (Table 6). The two QTLs (*KNcqSY-C1-1* and *KNcqSY-C1-2*) on C1 for SY were not observed in other genetic linkage groups, and these may be two new QTLs in the KN population.

**Table 6:** **Positions of homologous genes of ***Arabidopsis*** mapped on the KN linkage map and orthologous genes between ***B. napus*** and ***B. rapa/B. oleracea*****.

**Traits**	**Gene model**	***Arabidopsis* genes**	**Chr**.	**Gene position (cM)**	**QTLs**	**QTL position (cM)**	**CI. (cM)**	**Genes of *B. rapa/B. oleracea***	**Genes of *B. napus***
SY	*AT5G15230*	*GASA4*	A2	26.56				*Bra023513*	*BnaA02g02560D*
	*AT1G19670*	*ATCLH1*	A6	53.14				*Bra025756*	BnaA06g13830D
	*AT1G67090*	*RBCS1A*	C6	68.58					
	*AT1G75690*	*LQY1*	C6	71.37	*KNcqSY-C6-2*	70.06	65.64–77.08	*Bol027707*	BnaC06g36350D
	*AT1G78660*	*ATGGH1*	C6	77.08	*KNcqSY-C6-3*	76.14	65.64–77.08	*Bol027466*	BnaC06g39090D
SW	*AT5G10700*	*PTH2*	A2	3.16	*KNcqSW-A2-1*	34.31	0.0–44.0	*Bra006072*	
					*KNcqSW-A2-2*	37.61			
					*KNcqSW-A2-3*	43.01			
	*AT4G36920*	*AP2*	A3	98.68	*KNcqSW-A3-3*	104.15	92.8–113.3	*Bra011741*	*BnaA03g53830D*
	*AT1G73603*	*LCR64*	C6	71.37					
	*AT1G73607*	*LCR65*	C6	71.37	*KNcqSW-C6-2*,	67.2	65.16–78.92		
	*AT1G75830*	*PDF1*	C6	71.37	*KNcqSW-C6-3*	75.41	65.16–78.92	*Bol039878*	BnaC06g22110D
BY	*AT2G36450*	*HARDY*	A4	46.02	*KNcqBY-A4-1*	54.51	45.0–54.8	*Bra017235*	
PH	*AT5G23080*	*TGH*	A6	62.36			60.88–63.84	*Bra035958*	BnaC09g05000D
BH	*AT3G52430*	*ATPAD4*	A2	96.28			94.8–97.76	*Bra006922*	

*The QTL abbreviation “KNcq” represents consensus QTL from the KN Population*.

For SW, 40 QTLs were projected onto the KN consensus map (Figure 5, Figure S3, Table S7). *KNcqSW-A2-4* and *TNqSW-A2-3* were co-localized on A2. *KNcqSW-A3-3* and five QTLs of different populations were co-localized on A3, including two QTLs of TN (*TNqSW-A3-8* and *TNqSW-A3-9*) and three of BE (*BEcqSW-A3-2, BEcqSW-A3-3*, and *BEcqSW-A3-4*). The co-localized QTLs on KN and TN (*KNcqSW-C6-2, TNqSW-C6-3, KNcqSW-C6-3*, and *TNqSW-C6-2*) were also observed on the C6 linkage group (Table S8). A total of five orthologous genes for SW were observed: *PTH2, AP2, LCR64, LCR65*, and *PDF1* (Table 6). These results indicated that the QTLs for SW were reliable and reproducible.

For PH, 36 QTLs were projected onto the KN consensus map, including eight and nine onto A2 and A3, respectively (Figure 5, Figure S3, Table S7). *KNcqPH-A2* and two QTLs of TN (*TNqPH-A2-1* and *TNqPH-A2-2*) were co-localized on A2. *KNcqPH-A3-2* and three QTLs (*TNqPH-A3-1, BEqPH-A3-4*, and *BEqPH-A3-3*) were co-localized on A3. Likewise, *KNcqPH-A3-3* and *TNqPH-A3-4, KNcqPH-C3* and *QNqHPH-C3* were also co-localized on A3 and C3, respectively. In addition, *KNcqPH-C6-1* and *TNqPH-C6-3, KNcqPH-C6-2* and *TNqPH-C6-2* were co-localized in the same CI of C6, respectively. The QTLs on A7 (*KNcqPH-A7-1, KNcqPH-A7-2*, and *KNcqSW-A7-3*), A10 (*KNcqPH-A10*), C1 (*KNcqPH-C1*), C4 (*KNcqPH-C4*), and C9 (*KNcqPH-C9-1, KNcqPH-C9-2, KNcqPH-C9-3, KNcqPH-C9-4*, and *KNcqPH-C9-5*) were not observed in other genetic linkage groups. One orthologous gene *TGH* (*Bra035958*) on A6 was observed (Table 6), but no gene was aligned in the CI of QTLs for PH.

There were 34 QTLs for FBN projected onto the KN consensus map (Figure 5, Figure S3, Table S7), of which 10 were projected onto A3, and only two (*KNcqFBN-A3-2* and *BEqBN-A3-4*) were co-located (Table S8). Two QTLs were projected onto A6, of which *KNcqFBN-A6-3* and *TNqBN-A6*, and *KNcqFBN-A6-5* and *SEfb6.1* were co-localized. Three QTLs of KN (*KNcqFBN-C3-1, KNcqFBN-C3-2, KNcqFBN-C3-3*) and four QTLs of QN (*QNqHFB-C3-2, QNqHFB-C3-1, QNqFB-C3-1*, and *QNqFB-C3-2*) were co-localized. In addition, *KNcqFBN-C5* and *QNqFB-C5*, and *KNcqFBN-C6-1* and *TNqBN-C6-1* were co-localized on C5 and C6, respectively. Some potential new QTLs of the KN population were obtained on A2 (*KNcqFBN-A2*), A7 (*KNcqFBN-A7-1*), A10 (*KNcqFBN-A10-1* and *KNcqFBN-A10-2*), C2 (*KNcqFBN-C2*), C4 (*KNcqFBN-C4*), C7 (*KNcqFBN-C7*), and C9 (*KNcqFBN-C9-1* and *KNcqFBN-C9-2*).

The traits BY, BH, LMI, and PMI have rarely been studied in other populations, thus, no QTL for PMI was projected onto the KN consensus map. Due to lack of common markers between KN and other maps, only a few QTLs for BY, BH, LMI, and PMI from five populations were projected onto the KN consensus map, including six QTLs for BH and eight each for BY and LMI (Figure S3, Table S8), respectively. However, none of these QTLs were co-located with the QTLs in KN population. In addition, two orthologous genes were observed, including *HARDY* (*Bra017235*) for BY and *ATPAD4* (*Bra006922*) for BH (Table 6).

## Discussion

SY and SYRTs for *B. napus* are complex quantitative traits and easily affected by the environment (Quarrie et al., 2006). In previous studies, Chen et al. (2007) obtained 52 QTLs for six SYRTs and Fan et al. (2010) identified nine QTLs for SW. Chen et al. (2010) obtained 18 QTLs for SY and 22 QTLs for flowering time, while Butruille et al. (1999) found two QTLs for SY. These previous studies did not examine the relationship between SY and SYRTs, this was considered in the present study, showing correlation coefficients in the range of 0.31–0.83, except for FBN.

Several studies have revealed that only a few QTLs for SYRTs were stable in different environments (Li et al., 2007; Shi et al., 2009). The majority of consensus QTLs were also environment specific in the present study, including 70.8 and 23.6% of QTLs there were only detected in winter and spring areas, respectively. Only a few QTLs (5.6%) were detected in both areas, consistent with previous reports. These findings will be helpful for breeders to develop varieties with special adaptability.

Trait-by-trait meta-analysis revealed that 144 consensus QTLs for SY and SYRTs were integrated into 72 pleiotropic unique QTLs. Among them, six of seven unique QTLs for SY were co-located with two to five QTLs for SYRTs. On average, one unique QTL for SY involved 2.5 QTLs for SYRTs (Table 5). For example, *uq.A2-3* affected SY as well as five SYRTs: SW, BH, PH, FBN, and LMI. These findings were consistent with the strong correlations between SY and SYRTs. Shi et al. (2009) demonstrated that the QTLs for SY were pleiotropic and synthesized, and numerous SYRTs were potential contributor to tightly link with the QTLs for SY. Li et al. (2007) revealed that QTLs for SY and SYRTs usually had overlapping regions. Similar results were also reported for spring barley (Li et al., 2005) and red clover (Herrmann et al., 2006). The genes identified as having a function for SY and SYRTs also had pleiotropic effects for at least one trait (Ashikari et al., 2005; Lim et al., 2014) or multiple traits (Li et al., 1997; Hall et al., 2005; Quarrie et al., 2006; Burgess-Herbert et al., 2008; Hu et al., 2008). In other words, QTLs for SY and SYRTs might have resulted from pleiotropic QTLs that controlled multiple traits by containing multiple, closely linked, trait-specific genes (Hall et al., 2005).

Indicator QTLs have been successfully used in identifying genes with pleiotropic effects for SY (Shi et al., 2009). Indicator QTLs for SY must be stably expressed, easily measured, and identifiable candidate genes than the co-localized QTLs for SY. Using the similar methods, the candidate genes were successfully cloned by the QTL for BY in wheat (Quarrie et al., 2006), and the QTL for flowering time could be regarded as an indicator QTL for natural variation in rice (Xue et al., 2008). In the present study, four QTLs for SY closely co-existed with QTLs for SW (Table 5). SW can be more easily and precisely measured than SY, and is also less influenced by environmental factors than other SYRTs (Shi et al., 2009; Ding et al., 2012). Therefore, QTLs for SW could be regarded as indicator QTLs for SY. In future research, cloning the indicator QTL *KNcqSW-C6-3* for SY is more feasible than cloning *KNcqSY-C6-3*.

In the present study, 166 QTLs for SY and SYRTs from five populations were projected onto the constructed high-density consensus map. However, only 36 QTLs were co-located with QTLs identified in the KN population, including six QTLs for SY from the TN population and 11 for SW from the TN and BE population. Meanwhile, the co-located QTLs for SY were located on A2 and C6, while the co-located QTLs for SW were mainly located on A3 and C6. Thus, C6 was an important linkage group and included important genes for SY and SW. The reason that only a few QTLs were co-localized might be the lack of sufficient common markers between the different maps (Zhou et al., 2014) and also that there has been little research for some traits, such as PMI and LMI.

The known *Arabidopsis* genome sequence has been exploited as a tool for comparative analysis between *Arabidopsis* and *Brassicaceae* genomes, and the conserved genomic block has been identified in different *Brassicaceae* species (Arcade et al., 2004; Boivin et al., 2004; Schranz et al., 2006). This provided a method to align candidate genes underlying QTLs controlling important agronomic traits (Long et al., 2007). For SY, the orthologous gene *LQY1* was mapped on the C6 chromosome, which encodes a small zinc-finger-containing thylakoid membrane protein of *Arabidopsis* (Lu, 2011; Jin et al., 2014). Gene *LQY1* resulted in a lower quantum yield of photosystem II (PSII) photochemistry and reduced PSII electron transport rate following high-light treatment (Lu et al., 2011). For SW, gene *AP2* (*Bra011741* and *BnaA03g53830D*) and *PDF1* (*Bol039878* and *BnaC06g22110D*) were underlying the CIs of *KNcqSW-A3-3* and *KNcqSW-C6-3*, respectively. Gene *AP2* was involved in the specification of floral organ identity, establishment of floral meristem identity, ovule and seed coat development, and also had a role in controlling seed mass (Ohto et al., 2009). Gene *PDF1* is known as plant defense type 1 gene, and conferred high capacities to tolerate and hyperaccumulate zinc and cadmium (Mirouze et al., 2006). In this study, gene *PDF1* located on C6 controlled seed size and weight; however, the genetic mechanisms of controlling seed size and weight remained ambiguous. These genes are speculated to be candidate genes for SY or SYRTs in *B. napus*.

## Conclusion

The genetic mechanisms underlying SY and SYRTs were analyzed through QTL analysis in *B. napus*. A total of 226 QTLs were identified, and were integrated into 144 consensus QTLs. Seven major QTLs were obtained, including three QTLs for SY, two for SW and one each for BH and FBN, respectively. Trait-by-trait meta-analysis revealed that one unique QTL for SY involved 2.5 QTLs for SYRTs. Meanwhile, QTL projection from five different genetic linkage maps onto the KN consensus map showed that 36 QTLs were co-localized with QTLs identified in the KN population. In addition, candidate genes for SY and SYRTs were observed, including five each for SY (*GASA4, ATCLH1, RBCS1A, LQY1*, and *ATGGH1*) and SW (*PTH2, AP2, LCR64, LCR65*, and *PDF1*), and one each for BY (*HARDY*), BH (*ATPAD4*), and PH (*TGH*). The obtained candidate genes for SY and SYRTs were conducive to fine mapping and key gene cloning. These findings will be valuable in hybrid cultivar breeding and in analyzing QTL expression in different environments.

## Author contributions

WZ and XW carried out the QTL analysis and wrote the manuscript. JT, BL, LC, and HC participated in the field experiment. YL, JX, JG, and WL made helpful suggestions to the manuscript. HW and ML designed, led and coordinated the overall study.

## Funding

This work was supported financially by the National Science Foundation of China (31471532), the National Basic Research Program of China (2015CB150205), International Cooperation in Science and Technology Projects (2014DFA32210), the New Century Talents Support Program of the Ministry of Education of China (NCET110172), the Natural Science Foundation Research Key Projects of Shaanxi Province of China (2013JZ005) and International Cooperation Key Projects of Shaanxi Province of China (2012KW-15).

### Conflict of interest statement

The authors declare that the research was conducted in the absence of any commercial or financial relationships that could be construed as a potential conflict of interest.

## Abbreviations

B. napus: Brassica napus
B. rapa: Brassica rapa
B. oleracea: Brassica oleracea
QTL: Quantitative trait locus
DH: Doubled haploid
cM: CentiMorgan
CIs: Confidence intervals
PVE: Phenotypic variation explained
SY: Seed yield
SYRTs: Seed yield related traits
BY: Biomass yield
SW: Thousand seed weight
BH: First effective branch height
PH: Plant height
FBN: First effective branch number
LMI: Length of main inflorescence
PMI: Pod number of main inflorescence
LOD: Log odds score methods.

## References

[B1] Arcade A. Labourdette A. Falque M. Mangin B. Chardon F. Charcosset A.. (2004). BioMercator: integrating genetic maps and QTL towards discovery of candidate genes. Bioinformatics 20, 2324–2326. 10.1093/bioinformatics/bth230 15059820

[B2] Ashikari M. Sakakibara H. Lin S. Yamamoto T. Takashi T. Nishimura A.. (2005). Cytokinin oxidase regulates rice grain production. Science 309, 741–745. 10.1126/science.1113373 15976269

[B3] Basunanda P. Radoev M. Ecke W. Friedt W. Becker H. C. Snowdon R. J. (2010). Comparative mapping of quantitative trait loci involved in heterosis for seedling and yield traits in oilseed rape (*Brassica napus* L.). Theor. Appl. Genet. 120, 271–281. 10.1007/s00122-009-1133-z 19707740 PMC2793389

[B4] Boivin K. Acarkan A. Mbulu R. S. Clarenz O. Schmidt R. (2004). The Arabidopsis genome sequence as a tool for genome analysis in Brassicaceae. A comparison of the *Arabidopsis* and capsella rubella genomes. Plant Physiol. 135, 735–744. 10.1104/pp.104.040030 15208421 PMC514111

[B5] Burgess-Herbert S. Cox A. Tsaih S. W. Paigen B. (2008). Practical applications of the bioinformatics tool box for narrowing quantitative trait loci. Genetics 180, 2227–2235. 10.1534/genetics.108.090175 18845850 PMC2600954

[B6] Butruille D. V. Guries R. P. Osborn T. C. (1999). Linkage analysis of molecular markers and quantitative trait loci in populations of inbred backcross lines of *Brassica napus* L. Genetics 153, 949–964. 10511570 10.1093/genetics/153.2.949 PMC1460775

[B7] Cai D. Xiao Y. Yang W. Ye W. Wang B. Younas M.. (2014). Association mapping of six yield-related traits in rapeseed (*Brassica napus* L.). Theor. Appl. Genet. 127, 85–96. 10.1007/s00122-013-2203-9 24121524

[B8] Chen G. Geng J. Rahman M. Liu X. Tu J. Fu T. (2010). Identification of QTL for oil content, seed yield, and flowering time in oilseed rape (*Brassica napus*). Euphytica 175, 161–174. 10.1007/s10681-010-0144-9

[B9] Chen W. Zhang Y. Liu X. Chen B. Tu J. Fu T.. (2007). Detection of QTL for six yield-related traits in oilseed rape (*Brassica napus*) using DH and immortalized F_2_ populations. Theor. Appl. Genet. 115, 849–858. 10.1007/s00122-007-0613-2 17665168

[B10] Ding G. Liao Y. Yang M. Zhao Z. Shi L. Xu F. (2011). Development of gene-based markers from *Arabidopsis thaliana* functional genes involved in phosphorpus homeostasis and mapping in *Brassica napus*. Euphytica 181, 305–322. 10.1007/s10681-011-0428-8

[B11] Ding G. Zhao Z. Liao Y. Hu Y. Shi L. Long Y.. (2012). Quantitative trait loci for seed yield and yield-related traits, and their responses to reduced phosphorus supply in *Brassica napus*. Ann. Bot. 109, 747–759. 10.1093/aob/mcr323 22234558 PMC3286287

[B12] Fan C. Cai G. Qin J. Li Q. Yang M. Wu J.. (2010). Mapping of quantitative trait loci and development of allele-specific markers for seed weight in *Brassica napus*. Theor. Appl. Genet. 121, 1289–1301. 10.1007/s00122-010-1388-4 20574694

[B13] Hall M. C. Basten C. J. Willis J. H. (2005). Pleiotropic quantitative trait loci contribute to population divergence in traits associated with life-history variation in mimulus guttatus. Genetics 172, 1829–1844. 10.1534/genetics.105.051227 16361232 PMC1456280

[B14] Herrmann D. Boller B. Studer B. Widmer F. Kolliker R. (2006). QTL analysis of seed yield components in red clover (*Trifolium pratense* L.). Theor. Appl. Genet. 112, 536–545. 10.1007/s00122-005-0158-1 16331477

[B15] Hu K. Qiu D. Shen X. Li X. Wang S. (2008). Isolation and manipulation of quantitative trait loci for disease resistance in rice using a candidate gene approach. Mol. Plant 1, 786–793. 10.1093/mp/ssn039 19825581

[B16] Jiang C. Shi J. Li R. Long Y. Wang H. Li D.. (2014).Quantitative trait loci that control the oil content variation of rapeseed (*Brassica napus* L.). Theor. Appl. Genet. 127, 957–968. 10.1007/s00122-014-2271-5 24504552

[B17] Jiang C. Zeng Z. (1995). Multiple trait analysis of genetic mapping for quantitative trait loci. Genetics 140, 1111–1127. 7672582 10.1093/genetics/140.3.1111 PMC1206666

[B18] Jin H. Liu B. Luo L. Feng D. Wang P. Liu J.. (2014). Hypersensitive to high light1 interacts with low quantum yield of photosystem II1 and functions in protection of photosystem II from photodamage in *Arabidopsis*. Plant Cell 26, 1213–1229. 10.1105/tpc.113.122424 24632535 PMC4001379

[B19] Kearsey M. J. Pooni H. S. (1998). The Genetical Analysis of Quantitative Traits. Cheltenham: Stanley Thornes Publishers Ltd.

[B20] Li J. Huang X. Heinrichs F. Ganal M. W. Roder M. S. (2005). Analysis of QTLs for yield, yield components, and malting quality in a BC_3_-DH population of spring barley. Theor. Appl. Genet. 110, 356–363. 10.1007/s00122-004-1847-x 15549229

[B21] Li Y. Shen J. Wang T. Chen Q. Zhang X. Fu T. (2007). QTL analysis of yield-related traits and their association with functional markers in *Brassica napus* L. Aust. J. Agr. Res. 58, 759 10.1071/AR06350

[B22] Li Z. Pinson S. R. Park W. D. Paterson A. H. Stansel J. W. (1997). Epistasis for three grain yield components in rice (*Oryza sativa* L.). Genetics 145, 453–465. 9071598 10.1093/genetics/145.2.453 PMC1207809

[B23] Lim J. Yang H. Jung K. Yoo S. C. Paek N. C. (2014). Quantitative trait locus mapping and candidate gene analysis for plant architecture traits using whole genome re-sequencing in rice. Mol. Cells 37, 149–160. 10.14348/molcells.2014.2336 24599000 PMC3935628

[B24] Liu S. Liu Y. Yang X. Tong C. Edwards D. Parkin I. A.. (2014). The *Brassica oleracea* genome reveals the asymmetrical evolution of polyploid genomes. Nat. Commun. 5:3930. 10.1038/ncomms4930 24852848 PMC4279128

[B25] Lombard V. Delourme R. (2001). A consensus linkage map for rapeseed (*Brassica napus* L.): construction and integration of three individual maps from DH populations. Theor. Appl. Genet. 103, 491–507. 10.1007/s001220100560

[B26] Long Y. Shi J. Qiu D. Li R. Zhang C. Wang J.. (2007). Flowering time quantitative trait loci analysis of oilseed *Brassica* in multiple environments and genomewide alignment with *Arabidopsis*. Genetics 177, 2433–2444. 10.1534/genetics.107.080705 18073439 PMC2219480

[B27] Lu Y. (2011). The occurrence of a thylakoid-localized small zinc finger protein in land plants. Plant Signal. Behav. 6, 1881–1885. 10.4161/psb.6.12.18022 22080791 PMC3337170

[B28] Lu Y. Hall D. A. Last R. L. (2011). A small Zinc finger thylakoid protein plays a role in maintenance of Photosystem II in *Arabidopsis thaliana*. Plant Cell 23, 1861–1875. 10.1105/tpc.111.085456 21586683 PMC3123961

[B29] Maccaferri M. Sanguineti M. C. Corneti S. Ortega J. L. A. Salem M. B. Bort J.. (2008). Quantitative trait loci for grain yield and adaptation of durum wheat (*Triticum durum* Desf.) across a wide range of water availability. Genetics 178, 489–511. 10.1534/genetics.107.077297 18202390 PMC2206097

[B30] Mirouze M. Sels J. Richard O. Czernic P. Loubet S. Jacquier A.. (2006). A putative novel role for plant defensins: a defensin from the zinc hyper-accumulating plant, *Arabidopsis* halleri, confers zinc tolerance. Plant J. 47, 329–342. 10.1111/j.1365-313X.2006.02788.x 16792695

[B31] Ohto M. A. Floyd S. K. Fischer R. L. Goldberg R. B. Harada J. J. (2009). Effects of APETALA2 on embryo, endosperm, and seed coat development determine seed size in *Arabidopsis*. Sex. Plant Reprod. 22, 277–289. 10.1007/s00497-009-0116-1 20033449 PMC2796121

[B32] Paran I. Zamir D. (2003). Quantitative traits in plants: beyond the QTL. Trends Genet. 19, 303–306. 10.1016/S0168-9525(03)00117-3 12801720

[B33] Qiu D. Morgan C. Shi J. Long Y. Liu J. Li R.. (2006). A comparative linkage map of oilseed rape and its use for QTL analysis of seed oil and erucic acid content. Theor. Appl. Genet. 114, 67–80. 10.1007/s00122-006-0411-2 17033785

[B34] Quarrie S. Pekic Q. S. Radosevic R. Kaminska A. Barnes J. D. Leverington M.. (2006). Dissecting a wheat QTL for yield present in a range of environments: from the QTL to candidate genes. J. Exp. Bot. 57, 2627–2637. 10.1093/jxb/erl026 16831847

[B35] Quijada P. A. Udall J. A. Lambert B. Osborn T. C. (2006). Quantitative trait analysis of seed yield and other complex traits in hybrid spring rapeseed (*Brassica napus* L.): 1. Identification of genomic regions from winter germplasm. Theor. Appl. Genet. 113, 549–561. 10.1007/s00122-006-0323-1 16767447

[B36] Qzer H. Oral E. Dogru U. (1999). Relationships between yield and yield components on currently improved spring rapeseed cultivars. Tr. J. Agric. For. 53, 603–607.

[B37] Radoev M. Becker H. C. Ecke W. (2008). Genetic analysis of heterosis for yield and yield components in Rapeseed (*Brassica napus* L.) by quantitative trait locus mapping. Genetics 179, 1547–1558. 10.1534/genetics.108.089680 18562665 PMC2475754

[B38] Raman H. Raman R. Eckermann P. Coombes N. Manoli S. Zou X.. (2013). Genetic and physical mapping of flowering time loci in canola (*Brassica napus* L.). Theor. Appl. Genet. 126, 119–132. 10.1007/s00122-012-1966-8 22955939

[B39] Schranz M. E. Lysak M. A. Mitchell-Olds T. (2006). The ABC's of comparative genomics in the *Brassicaceae*: building blocks of crucifer genomes. Trends Plant Sci. 11, 535–542. 10.1016/j.tplants.2006.09.002 17029932

[B40] Scoles G. Fu F. Liu L. Chai Y. Chen L. Yang T.. (2007). Localization of QTLs for seed color using recombinant inbred lines of *Brassica napus* in different environments. Genome 50, 840–854. 10.1139/G07-068 17893725

[B41] Shi J. Li R. Qiu D. Jiang C. Long Y. Morgan C.. (2009). Unraveling the complex trait of crop yield with quantitative trait loci mapping in *Brassica napus*. Genetics 182, 851–861. 10.1534/genetics.109.101642 19414564 PMC2710164

[B42] Shi L. Shi T. Broadley M. R. White P. J. Long Y. Meng J.. (2013). High-throughput root phenotyping screens identify genetic loci associated with root architectural traits in *Brassica napus* under contrasting phosphate availabilities. Ann. Bot. 112, 381–389. 10.1093/aob/mcs245 23172414 PMC3698377

[B43] Udall J. A. Quijada P. A. Lambert B. Osborn T. C. (2006). Quantitative trait analysis of seed yield and other complex traits in hybrid spring rapeseed (*Brassica napus* L.): 2. Identification of alleles from unadapted germplasm. Theor. Appl. Genet. 113, 597–609. 10.1007/s00122-006-0324-0 16767446

[B44] UN (1935). Genome analysis in *Brassica* with special reference to the experimental formation of *B. napus* and peculiar mode of fertilization. Jpn. J. Bot. 7, 389–452.

[B45] Wang S. Basten C. Zeng Z. (2007). Windows QTL Cartographer 2.5. Raleigh, NC: North Carolina State University.

[B46] Wang X. Wang H. Long Y. Li D. Yin Y. Tian J.. (2013). Identification of QTLs associated with oil content in a high-oil *Brassica napus* cultivar and construction of a high-density consensus map for QTLs comparison in *B. napus*. PLoS ONE 8:e80569. 10.1371/journal.pone.0080569 24312482 PMC3846612

[B47] Wang X. Wang H. Long Y. Liu L. Zhao Y. Tian J.. (2015). Dynamic and comparative QTL analysis for plant height in different developmental stages of *Brassica napus* L. Theor. Appl. Genet. 128, 1175–1192. 10.1007/s00122-015-2498-9 25796183

[B48] Xue W. Xing Y. Weng X. Zhao Y. Tang W. Wang L.. (2008). Natural variation in *Ghd7* is an important regulator of heading date and yield potential in rice. Nat. Genet. 40, 761–767. 10.1038/ng.143 18454147

[B49] Yu F. (1998). Genetic analyses of several quantitative traits of doubled haploid population in *Brassica napus* L. (in Chinese with an English abstract). Sci. Agric. Sinica 31, 44–48.

[B50] Zhang G. He Y. Xu L. Tang G. Zhou W. (2006). Genetic analyses of agronomic and seed quality traits of doubled haploid population in *Brassica napus* through microspore culture. Euphytica 149, 169–177. 10.1007/s10681-005-9064-5

[B51] Zhou Q. Fu D. Mason A. Zeng Y. Zhao C. Huang Y. (2014). *In silico* integration of quantitative trait loci for seed yield and yield-related traits in *Brassica napus*. Mol. Breeding 33, 881–894. 10.1007/s11032-013-0002-2

