# Supplementary material for: Genome-Wide Identification of QTL for Seed Yield and Yield-Related Traits and Construction of a High-Density Consensus Map for QTL Comparison in Brassica napus
Source: Front Plant Sci. 2016 Jan 28;7:17. doi: 10.3389/fpls.2016.00017 (PMC4729939; doi:10.3389/fpls.2016.00017)
Supplement: Supplementary file 9 [file Image1.PDF]

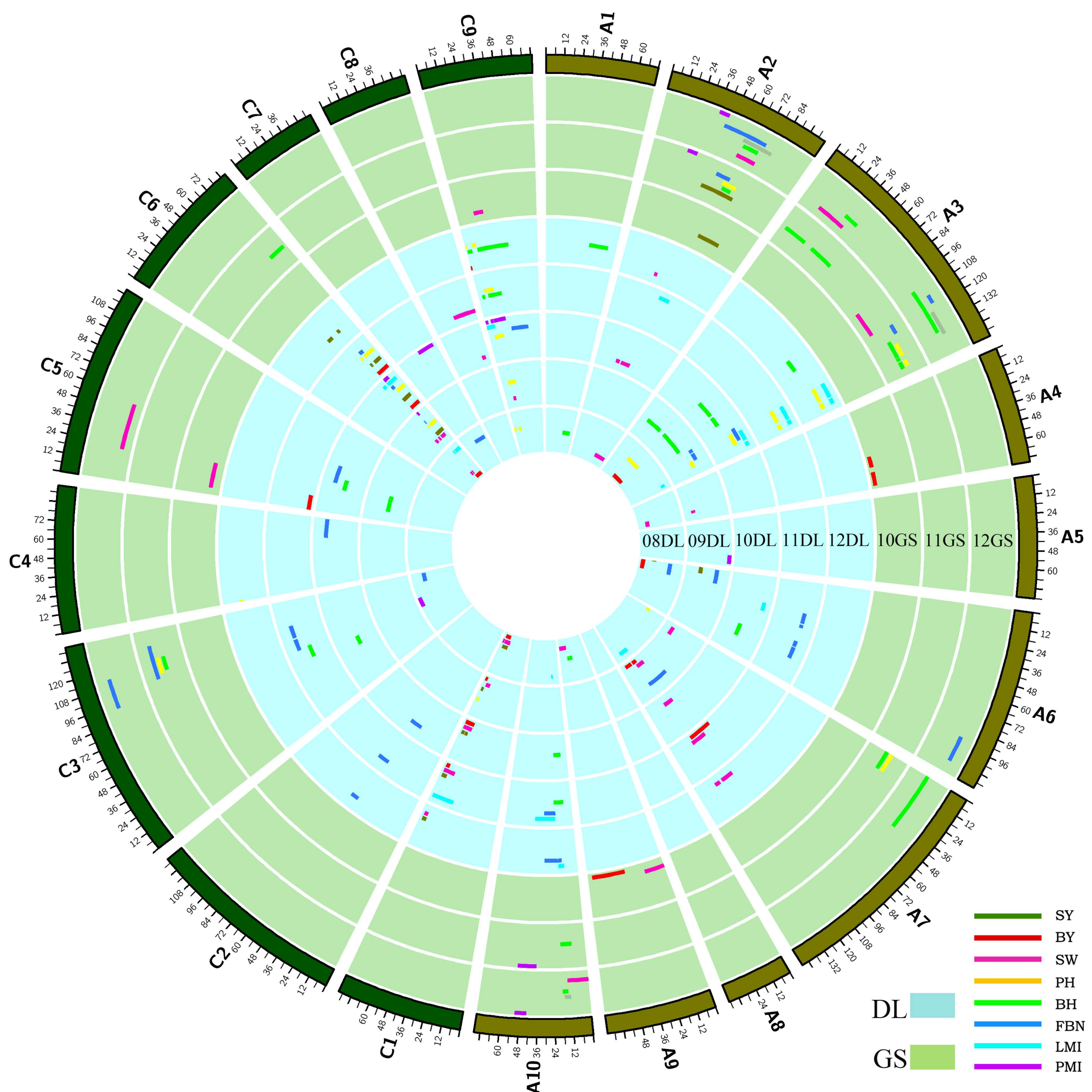

A1
A2
A3
A4
A5
A6
A7
A8
A9
A10
C1
C2
C3
C4
C5
C6
C7
C8
C9
08DL
09DL
10DL
11DL
12DL
10GS
11GS
12GS
DL
GS
SY
BY
SW
PH
BH
FBN
LMI
PMI

Figure S1: Expression response of consensus QTLs for SY and SYRTs from the KN population in natural environments. The QTLs distributed of 19 linkage groups in different natural environments were indicated by different backgrounds on the cycle (the backgrounds of wathet blue, the winter macro-environment; the backgrounds of light green, the spring macro-environment). Different environments and years were indicated by “years”+“location”, such as “09DL” indicated the winter macro-environment of the 2009 year and “09GS” indicated the spring macro-environment of the 2009 year. Grass green bar, SY (seed yield); Red bar, BY (biomass yield); Lavender bar, SW (thousand seed weight); Yellow bar, PH (plant height); Reseda bar, BH (first effective branch height); Blue bar, FBN (first effective branch number); Cambridge blue bar, LMI (length of main inflorescence); Purple bar, PMI (pod number of main inflorescence).
