# Supplementary material for: Genome-Wide Identification of QTL for Seed Yield and Yield-Related Traits and Construction of a High-Density Consensus Map for QTL Comparison in Brassica napus
Source: Front Plant Sci. 2016 Jan 28;7:17. doi: 10.3389/fpls.2016.00017 (PMC4729939; doi:10.3389/fpls.2016.00017)
Supplement: Supplementary file 10 [file Image2.PDF]

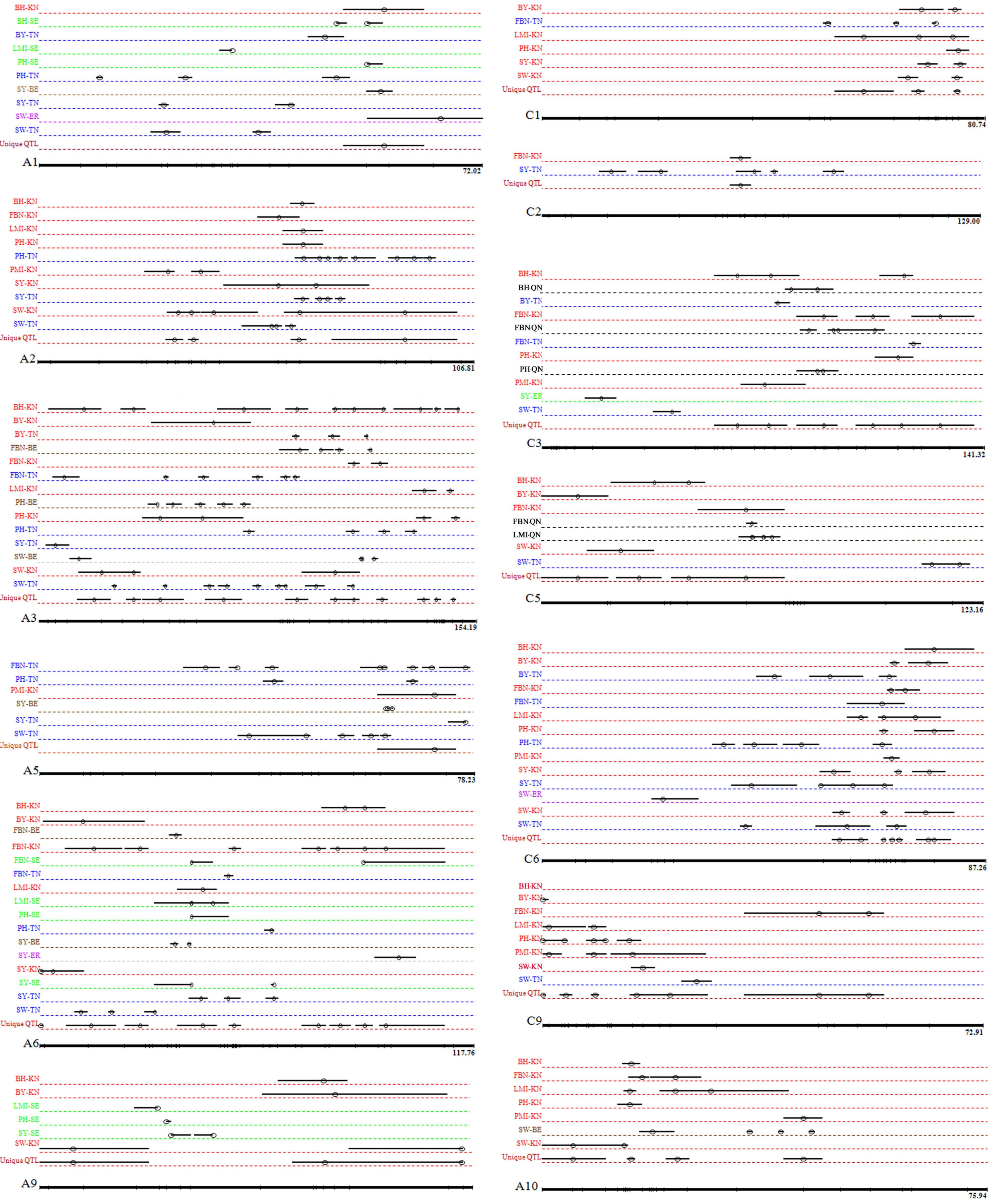
BH-KN
BH-SE
BY-TN
LMI-SE
PH-SE
PH-TN
SY-BE
SY-TN
SW-ER
SW-TN
Unique QTL
A1
72.02
BH-KN
FBN-KN
LMI-KN
PH-KN
PH-TN
PMI-KN
SY-KN
SY-TN
SW-KN
SW-TN
Unique QTL
A2
106.81
BH-KN
BY-KN
BY-TN
FBN-BE
FBN-KN
FBN-TN
LMI-KN
PH-BE
PH-KN
PH-TN
SY-TN
SW-BE
SW-KN
SW-TN
Unique QTL
A3
154.19
FBN-TN
PH-TN
PMI-KN
SY-BE
SY-TN
SW-TN
Unique QTL
A5
78.23
BH-KN
BY-KN
FBN-BE
FBN-KN
FBN-SE
FBN-TN
LMI-KN
LMI-SE
PH-SE
PH-TN
SY-BE
SY-ER
SY-KN
SY-SE
SY-TN
SW-TN
Unique QTL
A6
117.76
BH-KN
BY-KN
LMI-SE
PH-SE
SY-SE
SW-KN
Unique QTL
A9
BY-KN
FBN-TN
LMI-KN
PH-KN
SY-KN
SW-KN
Unique QTL
C1
80.74
FBN-KN
SY-TN
Unique QTL
C2
129.00
BH-KN
BH-QN
BY-TN
FBN-KN
FBNQN
FBN-TN
PH-KN
PH-QN
PMI-KN
SY-ER
SW-TN
Unique QTL
C3
141.32
BH-KN
BY-KN
FBN-KN
FBNQN
LMI-QN
SW-KN
SW-TN
Unique QTL
C5
123.16
BH-KN
BY-KN
BY-TN
FBN-KN
FBN-TN
LMI-KN
PH-KN
PH-TN
PMI-KN
SY-KN
SY-TN
SW-ER
SW-KN
SW-TN
Unique QTL
C6
87.26
BH-KN
BY-KN
FBN-KN
LMI-KN
PH-KN
PMI-KN
SW-KN
SW-TN
Unique QTL
C9
72.91
BH-KN
FBN-KN
LMI-KN
PH-KN
PMI-KN
SW-BE
SW-KN
Unique QTL
A10
75.94

Figure S2: **Distribution of unique QTL in KN population and consensus QTL for SY and SYRTs on KN linkage groups from the KN and different populations.** Whole linkage groups were shown with black lines and labeled with molecular markers (short vertical bars) on the bottom, and the Arabic numbers lists on the right show the length of linkage groups. The names of traits and populations were listed on the left side of the linkage groups, such as “SY-KN” were indicated the QTL for SY from the KN population. The black lines on the linkage groups show the QTL confidence interval and the cycles indicate the peak position.
