# Supplementary material for: Genome-Wide Identification of QTL for Seed Yield and Yield-Related Traits and Construction of a High-Density Consensus Map for QTL Comparison in Brassica napus
Source: Front Plant Sci. 2016 Jan 28;7:17. doi: 10.3389/fpls.2016.00017 (PMC4729939; doi:10.3389/fpls.2016.00017)
Supplement: Supplementary file 11 [file Image3.PDF]

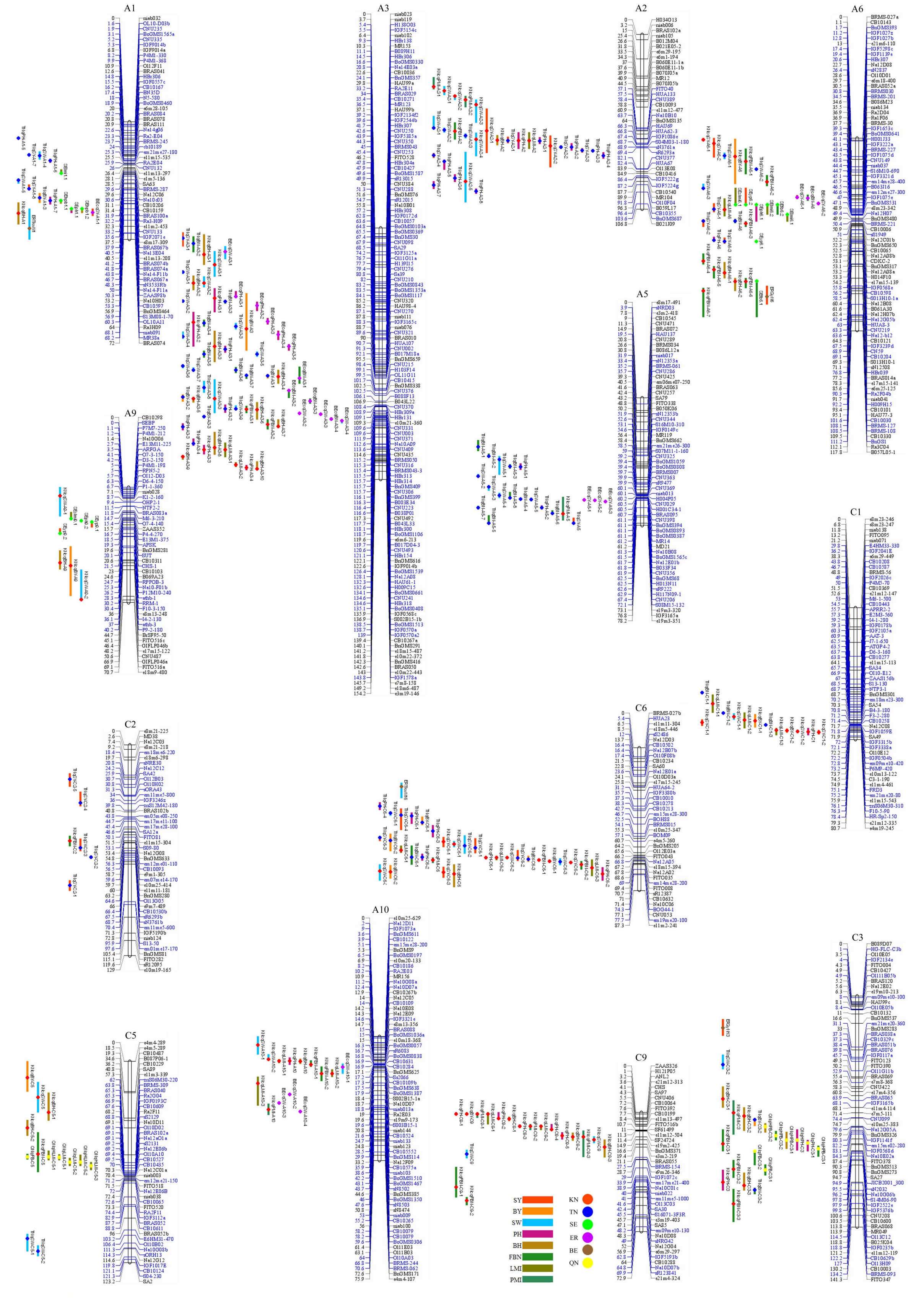
A1
A3
A2
A6
A5
A9
C1
C2
C6
C5
A10
C3
C9
SY
BY
SW
PH
BH
FBN
LMI
PMI
KN
TN
SE
ER
BE
QN

Figure S3: The consensus map and QTLs for SY and SYRTs projected in different populations. Markers with black color indicated these markers were on the KN genetic map and blue color indicated these makers were projected from other maps on the KN map. “The name of population”+“cq or q” indicated the QTL for SY and SYRTS from different populations, for example, *KNcqSY-A2-1* indicated the QTLs from the KN population and SY indicated the trait of seed yield. The QTLs for KN population projected in five populations were indicated by bars with various backgrounds on the left of each linkage group. Red bar, SY (seed yield); Orange bar, BY (biomass yield); Cambridge blue bar, SW (thousand seed weight); Purple bar, PH (plant height); Claybank bar, BH (first effective branch height); Green bar, FBN (first effective branch number); Breen bar, LMI (length of main inflorescence); Blue-green bar, PMI (pod number of main inflorescence). The QTLs from the KN population and five populations were indicated by disks with various backgrounds on the bars of each QTL. Red disk, KN population; Blue disk, TN population; Light green disk, SE population; Light purple disk, ER population; Brown disk, BE population; Yellow disk, QN population.
